# CEST MRI provides amide/amine surrogate biomarkers for treatment-naïve glioma sub-typing

**DOI:** 10.1007/s00259-022-05676-1

**Published:** 2022-01-14

**Authors:** Laura Mancini, Stefano Casagranda, Guillaume Gautier, Philippe Peter, Bruno Lopez, Lewis Thorne, Andrew McEvoy, Anna Miserocchi, George Samandouras, Neil Kitchen, Sebastian Brandner, Enrico De Vita, Francisco Torrealdea, Marilena Rega, Benjamin Schmitt, Patrick Liebig, Eser Sanverdi, Xavier Golay, Sotirios Bisdas

**Affiliations:** 1 Box65, Lysholm Department of Neuroradiology, The National Hospital for Neurology & Neurosurgery, University College London Hospitals NHS Foundation Trust, 8-11 Queen Square, London, WC1N 3BG UK; 2 Department of Brain Repair and Rehabilitation, UCL Queen Square Institute of Neurology, London, UK; 3 Olea Medical, La Ciotat, France; 4 Department of Neurosurgery, The National Hospital for Neurology & Neurosurgery, University College London Hospitals NHS Foundation Trust, London, UK; 5 Division of Neuropathology, UCL Queen Square Institute of Neurology, London, UK; 6 The National Hospital for Neurology and Neurosurgery, University College London Hospitals NHS Foundation Trust, London, UK; 7 University College Hospital, University College of London Hospitals NHS Foundation Trust, London, UK; 8 Siemens Healthineers, Erlangen, Germany

**Keywords:** Isocitrate dehydrogenase, 1p/19q codeletion, Oligodendroglioma, Chemical exchange saturation transfer, Glioma risk stratification

## Abstract

**Purpose:**

Accurate glioma classification affects patient management and is challenging on non- or low-enhancing gliomas. This study investigated the clinical value of different chemical exchange saturation transfer (CEST) metrics for glioma classification and assessed the diagnostic effect of the presence of abundant fluid in glioma subpopulations.

**Methods:**

Forty-five treatment-naïve glioma patients with known isocitrate dehydrogenase (IDH) mutation and 1p/19q codeletion status received CEST MRI (*B*_1rms_ = 2μT, *T*_sat_ = 3.5 s) at 3 T. Magnetization transfer ratio asymmetry and CEST metrics (amides: offset range 3–4 ppm, amines: 1.5–2.5 ppm, amide/amine ratio) were calculated with two models: ‘asymmetry-based’ (AB) and ‘fluid-suppressed’ (FS). The presence of T2/FLAIR mismatch was noted.

**Results:**

IDH-wild type had higher amide/amine ratio than IDH-mutant_1p/19q^codel^ (*p* < 0.022). Amide/amine ratio and amine levels differentiated IDH-wild type from IDH-mutant (*p* < 0.0045) and from IDH-mutant_1p/19q^ret^ (*p* < 0.021). IDH-mutant_1p/19q^ret^ had higher amides and amines than IDH-mutant_1p/19q^codel^ (*p* < 0.035). IDH-mutant_1p/19q^ret^ with AB/FS mismatch had higher amines than IDH-mutant_1p/19q^ret^ without AB/FS mismatch ( < 0.016). In IDH-mutant_1p/19q^ret^, the presence of AB/FS mismatch was closely related to the presence of T2/FLAIR mismatch (*p* = 0.014).

**Conclusions:**

CEST-derived biomarkers for amides, amines, and their ratio can help with histomolecular staging in gliomas without intense contrast enhancement. T2/FLAIR mismatch is reflected in the presence of AB/FS CEST mismatch. The AB/FS CEST mismatch identifies glioma subgroups that may have prognostic and clinical relevance.

**Supplementary Information:**

The online version contains supplementary material available at 10.1007/s00259-022-05676-1.

## Introduction

The discovery of mutations in the IDH1 and IDH2 genes in astrocytic and oligodendroglial tumours has led to a biomarker-driven classification, forming an integrated diagnosis composed of the histological appearance and the molecular profile [1].

Currently, two types of IDH-mutant gliomas are identified. The IDH-mutant astrocytoma is defined by an additional mutation of ATRX and p53. A loss in the tumour suppressor locus CDKN2A/B is an important additional prognostic marker, typically found in IDH-mutant glioblastomas. The IDH-mutant oligodendroglioma is defined by an absent ATRX mutation, and a codeletion of chromosomal arms 1p and 19q. IDH-mutant oligodendrogliomas consistently carry a TERT promoter mutation. The IDH-wild-type gliomas comprise a wide range of tumours, including, but by far not limited to, the IDH-wild-type glioblastoma. The IDH-wild-type glioblastoma is molecularly characterized by chromosome 7 gain, chromosome 10 loss, frequent EGFR amplification, and TERT promoter mutation. However, there is a wide range of other IDH-wild-type gliomas of low and high grades, with other defining mutations, such as BRAF, histone H3 K27M, and H3 G34R.

Several advanced MRIs exploiting different tissue compositions and properties have been shown to add diagnostic and prognostic value to the conventional gadolinium-enhanced MRI protocols, which cannot reliably sample the genetic makeup of the tumours. Examples are diffusion-weighted imaging (DWI) [2–6]; perfusion-weighted imaging [7–10]; magnetic resonance spectroscopy (MRS) [11, 12]; and, recently, chemical exchange saturation transfer (CEST). CEST MRI is based on proton-exchange properties and allows imaging of low-concentration metabolites (concentrations in vivo down to mM range) with enhanced sensitivity indirectly through the water signal [13, 14]. The CEST MRI signal detectable from amide protons (–NH groups resonating at 3.5 ppm downfield from the water peak) and of the amine protons (–NH_2_ groups, at 2 ppm), present in endogenous proteins and peptides, has been shown in small patient cohorts and feasibility studies to differentiate low-grade gliomas from high-grade gliomas [15–17] with better diagnostic performance than diffusion- and perfusion-weighted imaging [18–20]; tissue heterogeneities in high-grade gliomas [21]; tumour progression from radiation necrosis [22, 23]; IDH-wild type from IDH-mutant glioma [24]; and IDH-mutant with 1p/19q codeletion from 1p/19q-intact IDH-mutant glioma [24, 25]. CEST-derived metrics have also been shown to correlate with patient overall survival and progression-free survival [26–28].

The current evidence for the clinical utility of CEST metrics has inherent study design and methodological weaknesses. The former includes mainly the small number of patients; the unbalanced cohort composition with diagnostically straightforward, by means of conventional MRI, high-grade gliomas; and the use of the outdated WHO 2007 classification for glioma grading in the prevailing number of studies. Methodological issues are related to the investigation only of the 3.5 ppm amide frequency and negligence of the T2/FLAIR mismatch effect on the CEST maps, which carries significant diagnostic implications as CEST is sensitive to fluid signal [29]. Studies in rats and phantoms have suggested that the CEST contribution at 2 ppm is sensitive to proteins, amino acid, and pH concentration changes, and may serve as a sensitive neuroimaging biomarker for many diseases [30], while a study in vitro and on mice has suggested the amide/amine ratio to be sensitive to the tissue acidity in stroke [31].

The current study sought to address the knowledge gap on the clinical value of CEST imaging to predict the histomolecular glioma type by prospectively investigating the whole range of frequency offsets either side of the water peak from 0 to 4 ppm in a single-centre setting, large patient cohort presenting mainly with the diagnostically challenging primary gliomas with absent or weak enhancement. For the first time, we sought to evaluate the diagnostic value of tumour amide/amine ratio, which might be a promising surrogate biomarker for gliomas sub-typing. We also aimed to assess the diagnostic sensitivity and specificity of two different CEST models (‘asymmetry-based’ and ‘fluid-suppressed’) to address any diagnostic compromise caused by the presence of abundant fluid in gliomas subpopulations, i.e. those with T2/FLAIR mismatch.

## Materials and methods

### Patients

**Fig. 1:**
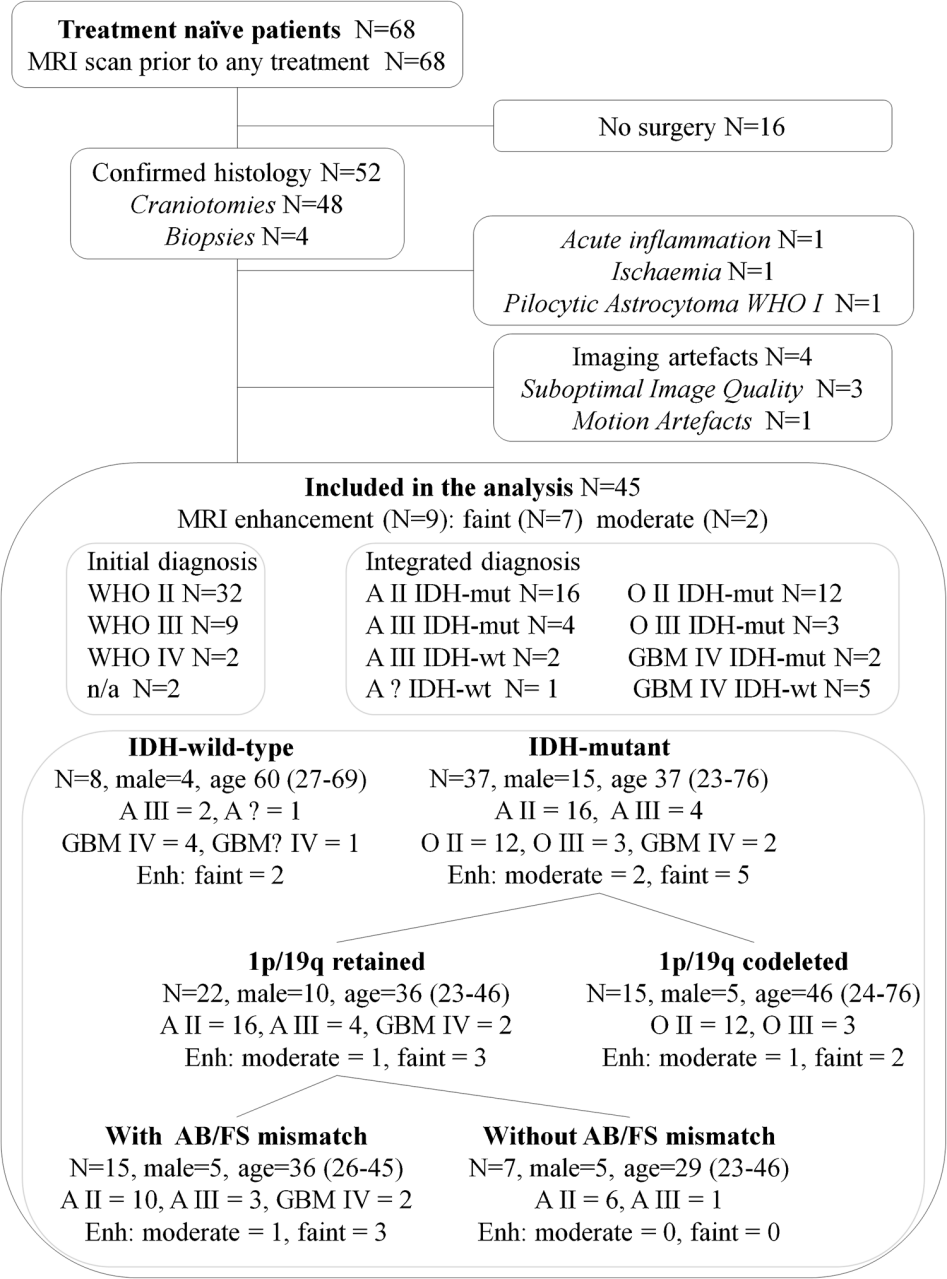
Patient recruitment and histology diagram. Forty-five histologically proven glioma patients were included in this prospective study. All patients had an MRI scan prior to receiving any treatment. Surgery (craniotomies or biopsies) occurred a median (range) of 1.8 (0.1–30.7) months after MRI. A = astrocytoma, O = oligodendroglioma, GBM = glioblastoma; II, III, IV = WHO grades. Enh = subjects with post-gadolinium enhancement. Age is in years = median (range)

Treatment-naïve patients, older than 18 years of age, with presumed glioma suitable for surgical lesion sampling or resection were selected during the weekly held multidisciplinary tumour board meeting by unanimous agreement among the clinical and neuroradiology board members. Exclusion criteria included usual contraindications to MRI, pregnancy, and incapacity to provide informed consent. Eighty-two patients have been so far successfully recruited for the study. For the objectives of this publication, an interim study analysis of 68 patients with conventional indeterminate MRI findings for glioma staging was conducted (Fig. 1). After the MRI, 48 patients underwent surgical resection and four had a biopsy. Of these, 49 patients had histologically confirmed gliomas with grade II or higher and two patients had acute inflammation and multifocal cortical ischaemia, respectively. Data from four patients were discarded due to presence of artefacts. The MRIs of nine out of 45 patients showed post-contrast enhancement, of which seven were described as faint enhancement and two as moderate enhancement. Finally, 45 patients (19 male), who had undergone surgery/biopsy a median (range) of 1.8 (0.1–30.7) months after MRI, were included in the analysis. The clinical characteristics of the patient population are summarized in Fig. 1 and detailed in Supplementary Table 1.

### MRI data acquisition

Imaging data were acquired on a 3-T whole-body MRI system (MAGNETOM Prisma; Siemens Healthcare, Erlangen, Germany) with a 64-channel Head/Neck coil. Structural T_1_-weighted (T_1_w), T_2_w, fluid-attenuated inversion recovery (FLAIR), and gadolinium-enhanced T_1_w (Gd-T_1_w) images were acquired with sagittal 3D Sampling Perfection with Application optimized Contrasts using different flip angle Evolution (SPACE) images, with an isotropic resolution of 0.9 × 0.9 × 0.9 mm^3^, 1.1 × 1.1 × 1.1 mm^3^, 1.0 × 1.0 × 1.0 mm^3^, and 0.9 × 0.9 × 0.9 mm^3^, respectively. The axial single-slice prototype 2D CEST sequence (1.6 × 1.6 × 4.0 mm^3^) was located at the largest tumour cross-section. Three acquisitions with different saturation pulse intensities (*B*_1rms_ = 1.7μT, 2μT, 2.3μT; *T*_sat_ = 3.5 s) were performed. A relative *B*_1_ map [32] was obtained by a dual flip angle method for *B*_1_-correction of the CEST signal. The total acquisition time was approximately 17 min for the structural data and 8 min for the CEST data. Detailed description of the conventional MRI protocol and the CEST acquisition is provided in the Supplementary Material.

### Regions of interest

Regions of interest (ROIs) were manually drawn by two senior neuroradiologists (12 and 8 years of experience in neuro-oncology imaging) in consensus using ITK-SNAP version 3.6 (www.itksnap.org) [33] loading first the CEST to identify the appropriate slice position and then the co-registered FLAIR, T2w, T1w, and Gd-T1w images. Areas identified were (1) solid tumour excluding cyst and necrotic areas, (2) cyst, and (3) normal appearing white matter (NAWM) contralateral to the tumour.

### CEST post-processing

CEST acquisitions were processed using prototype version software created by Olea Medical® (La Ciotat, France) for the Horizon 2020 project GLINT (number 667510). After Z-Spectra-based B_0_ correction [34] of the three acquired *B*_1_ saturation levels, the Z-Spectra at 2.0μT were *B*_1_-corrected through an exponential fitting [32]. Magnetization transfer ratio asymmetry (MTR_asym_) spectra and proton transfer-weighted maps with two different models and in two offset ranges were then calculated.

### *MTR asymmetry spectra and ∆MTR*_*asym*_* spectra*

MTR_asym_ spectra were calculated at steps of 0.25 ppm from 0 to 4 ppm with a previously reported formula [35]. Changes in MTR_asym_ (∆MTR_asym_) were obtained subtracting the NAWM MTR_asym_ from the tumour one: ∆MTR_asym_ (∆ω) = MTR_asym_ (∆ω, tumour) − MTR_asym_ (∆ω, NAWM). Average and 25th and 75th percentiles of the MTR_asym_ and of the ∆MTR_asym_ spectra from patients in the various histological groups were calculated to generate the corresponding group spectra and group range of variation.

### *Asymmetry-based and fluid-suppressed APT*_*w*_* image processing*

Non-punctual metrics [36] obtained averaging the signal in a 1-ppm range centred at 3.5 ppm and at 2 ppm were adapted to generate two amide and amine proton transfer-weighted maps (APT_w_ (∆3.5) and APT_w_ (∆2), respectively). These had improved detection (with respect to the punctual metrics at 3.5 ppm and 2 ppm) of the CEST contrast for *B*_0_- and *B*_1_-corrected Z-Spectra at 2.0μT. APT_w_ maps were obtained using an asymmetry-based (AB_APT_w_) model and a fluid-suppressed (FS_APT_w_) model [29]. The AB_APT_w_ considered only the asymmetry-average of the Z-Spectra while the FS_APT_w_ also attenuated the fluid signal based on the shape of Z-spectrum, as described in the Supplementary Material. Furthermore, the difference (mismatch) between AB and FS models was evaluated in terms of AB-FS maps.

The endogenous CEST signal was explored in two offset ranges: amines (∆2, from 1.5 to 2.5 ppm) and amides (∆3.5, from 3.0 to 4.0 ppm), obtaining four maps. In total, nine normalized metrics (obtained by subtracting the average signal in the contralateral NAWM ROI from the average values in the tumour ROI) were considered: the amides, amines, and the amide/amine ratios for each of the AB, FS, and AB-FS maps.

### T2/FLAIR mismatch vs AB/FS mismatch in IDH-mutant gliomas

The T2 and FLAIR images were assessed in consensus by a senior neuroradiologist (14 years of experience as consultant neuroradiologist with expertise in neuro-oncology) and an MRI physicist (15 years of experience in MRI image analysis) for identification of the presence of T2/FLAIR mismatch. The T2/FLAIR mismatch was defined as the presence of > 50% hyperintense signal on T_2_w and relatively hypointense signal on FLAIR except for a hyperintense peripheral rim in cases of complete or near-complete mismatch [37, 38].

Cut points were calculated (method outlined in the ‘Statistical analysis’ section) for the AB-FS (∆3.5) and AB-FS (∆2) metrics in order to assess if AB-FS metrics could serve as surrogate biomarkers for the presence/absence of T2/FLAIR mismatch (reference standard). The diagnostic value of AB/FS mismatch, defined as AB-FS values larger than the cut point threshold, was assessed.

### Histopathology

Tumour biopsies were fixed in formalin and processed into paraffin-embedded samples. Tissue sections were routinely stained with haematoxylin and eosin, and immunostained with antibodies against IDH1 R132H, ATRX, and Ki67. IDH1 R132H-positive and ATRX-negative tumours (loss of expression) were diagnosed as IDH-mutant astrocytoma, anaplastic astrocytoma, or glioblastoma, depending on the histological features. IDH1 R132H-positive and ATRX-positive (retained) tumours were examined for the presence of a 1p/19q codeletion (1p/19q^ret^ or 1p/19q^codel^). All IDH1 R132H-negative tumours were further sequenced for the presence of a rare IDH1 or an IDH2 mutation. These tumours were also tested for TERT promoter mutation and EGFR amplification to identify the population of IDH-wild-type glioblastoma. Tumours that had no informative molecular profile were further examined with DNA methylation arrays, followed by algorithmic classification as described in [39].

### Statistical analysis

Following a Shapiro-Wilk normality test, the asymmetry-based (AB) and fluid-suppressed (FS) CEST metrics were compared using a sign test with two-tailed *p*-values and significance threshold set to 0.05. A Fisher's exact test assessed the relationship between the presence/absence of AB/FS mismatch and the presence/absence of T2/FLAIR mismatch.

The Mann-Whitney *U* test was used to analyze the statistical differences between quantitative imaging parameters for the two IDH mutation statuses. The Kruskal-Wallis test followed, when significant, by the Conover-Iman test to correct for multiple pairwise comparison (significance set to 0.05), was used to assess the statistical differences for (i) the three glioma groups IDH-wild type, IDH-mutant_1p/19q^ret^, and IDH-mutant_1p/19q^codel^; and (ii) the four tumour subgroups IDH-wild type, IDH-mutant_1p/19q^ret^ with (without) AB/FS mismatch, and IDH-mutant_1p/19q^codel^. For pairs of groups with statistically significant differences after correction for multiple comparisons, areas under the receiver operating characteristic (ROC) curve (AUC) and the nearest to (0.1) cut points were estimated. All statistical analyses were performed with Stata software (StataCorp. 2017. Stata Statistical Software: Release 15. College Station, TX: StataCorp LLC).

## Results

### *Z-Spectra, APT*_*w*_* maps, and MTR*_*asym*_* and ∆MTR*_*asym*_* spectra*

**Fig. 2:**
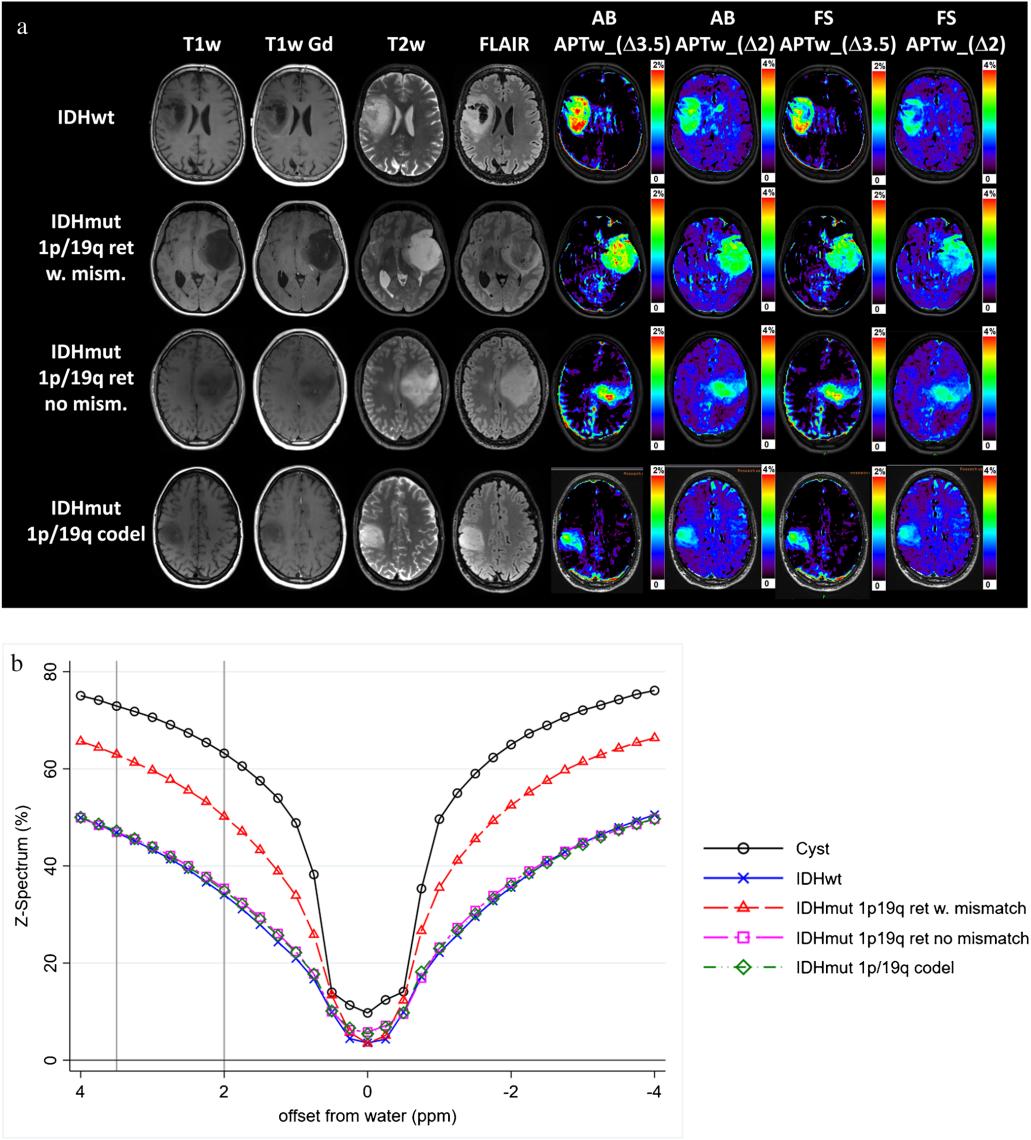
Representative examples of structural MRI, CEST maps, and Z-spectra. **a** Representative structural MRI and CEST maps for IDH-wild-type, IDH-mutant_1p/19q^ret^ with and without AB/FS mismatch, and IDH-mutant_1p/19q^codel^. AB_APT_w_ indicates the asymmetry-based metric while FS_APT_w_ is the fluid-suppressed metric. ∆3.5 is the 1-ppm offset range centred at the amide offset of 3.5 ppm, while ∆2 is the 1-ppm offset range centred at the amine offset of 2 ppm. **b** Representative Z-spectra of the cyst component and of the tumour core for the same tumour types

**Fig. 3:**
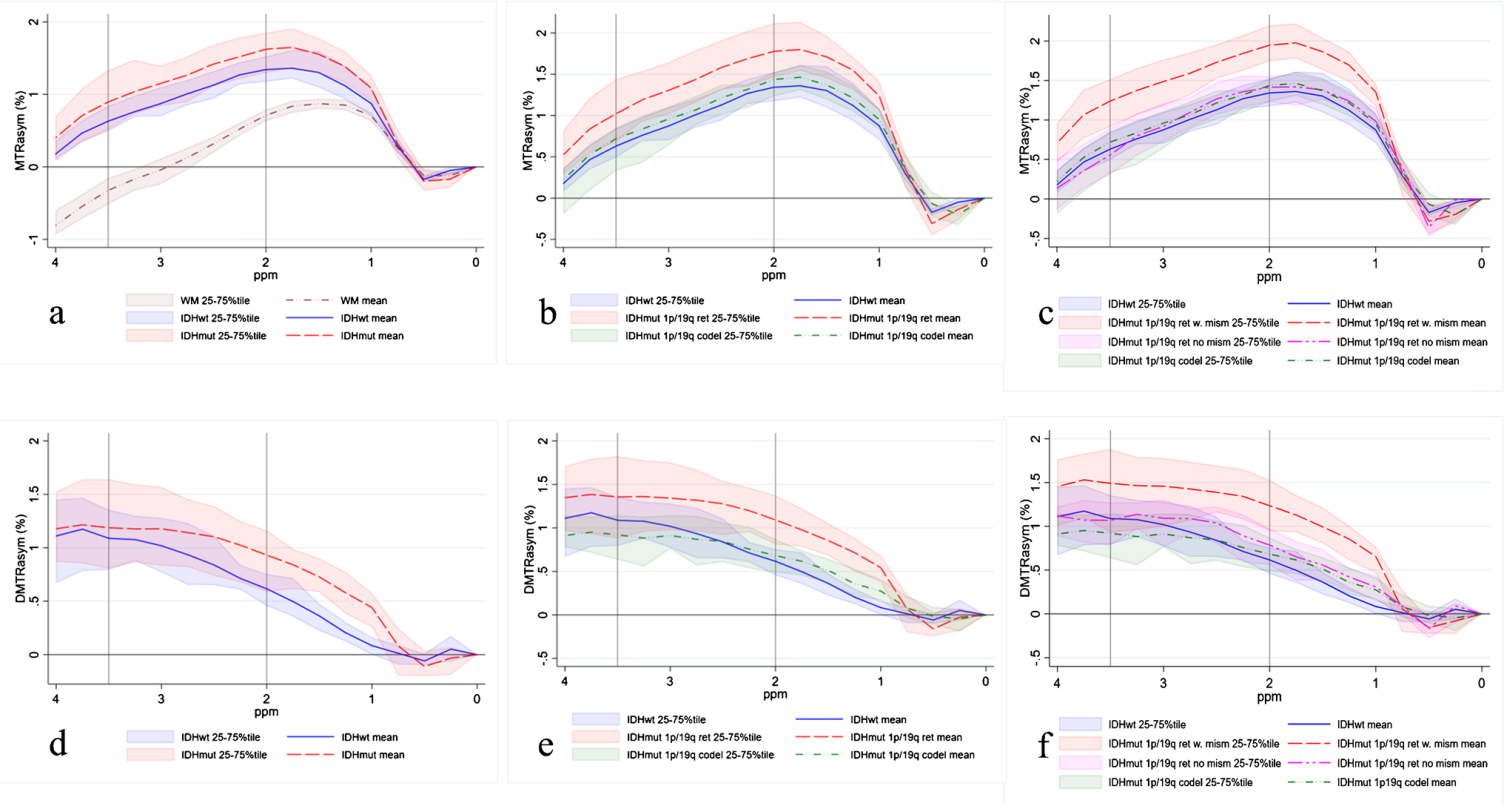
Comparison of the average MTR_asym_ spectra (**a**, **b**, **c**) and average ∆MTR_asym_ spectra (**d**, **e**, **f**). The lines represent average values over tumour types. The shaded areas represent the range of variation between the 25th percentile and the 75th percentile. The vertical lines at 2 ppm and 3.5 ppm indicate the center of the amine and of the amide regions. **a**, **d** IDH-wild-type vs IDH-mutant; **b**, **e** IDH-wild-type vs IDH-mutant_1p/19q^ret^ vs IDH-mutant_1p/19q^codel^; **c**, **f** IDH-wild-type vs IDH-mutant_1p/19q^ret^ with AB/FS mismatch vs IDH-mutant_1p/19q^ret^ without AB/FS mismatch vs IDH-mutant_1p/19q^codel^. In **a**, the average MTR_asym_ spectrum of the contralateral normal appearing white matter (NAWM) is also shown, averaged over all patients

Representative examples for four different tumour types of structural MRIs and CEST maps (shown in Fig. 2a) and Z-Spectra (shown in Fig. 2b) show a cystic component present in the IDH-wild-type with reduced signal in the FS CEST maps in the amide and amine ranges (Fig. 2a, IDH-wild-type); minor differences between AB and FS maps in the solid tumour, more evident in IDH-mutant_1p/1q^ret^ and in the amine range (Fig. 2a). Fig. 3 shows the MTR_asym_ (Fig. 3a–c) and the ∆MTR_asym_ spectra (Fig. 3d–f) for the different tumour subtypes (two glioma groups in Fig. 3a and d; three glioma groups in Fig. 3b and e; four glioma groups in Fig. 3c and f). Both the MTR_asym_ and the ∆MTR_asym_ spectra showed different contributions from the amide and the amine regions between glioma subtypes over the frequency range analyzed. The average MTR_asym_ spectrum in the NAWM over all patients was null at a frequency of ~ 3 ppm from the water resonance (Fig. 3a).

### CEST metrics

**Fig. 4:**
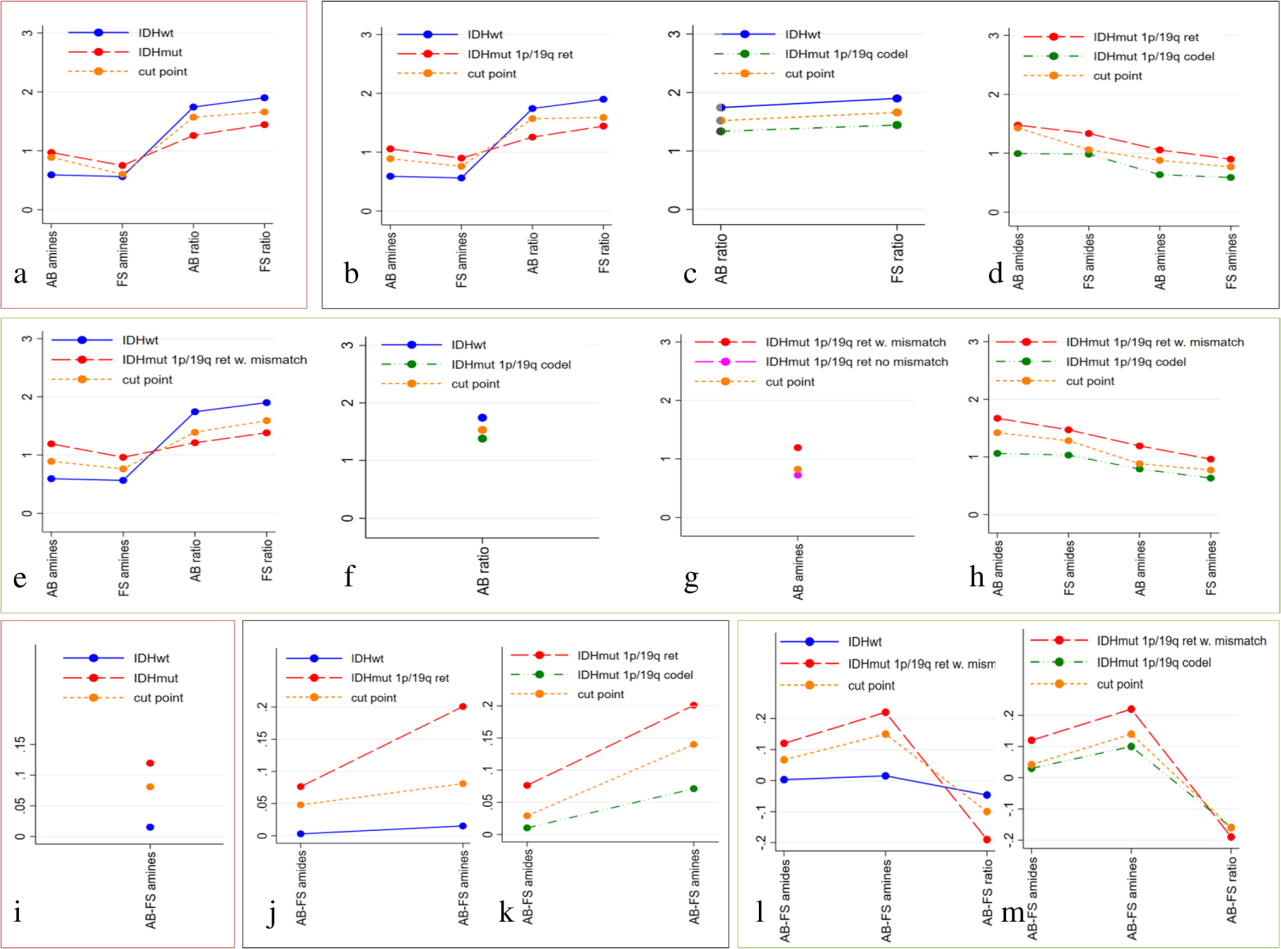
Pairwise comparison of groups with statistically significant differences in the represented metrics. The medians of the metrics are depicted, together with the cut points. **a–h** The AB and AF metrics. **i–m** The AB-FS metrics. **a** and **i** IDH-wild-type vs IDH-mutant. **b**–**d** and **j**–**k** Three tumour types: IDH-wild-type; IDH-mutant_1p/19q^ret^; IDH-mutant_1p/19q^codel^. **e**–**h** and **l**–**m** Four tumour types: IDH-wild-type; IDH-mutant_1p/19q^ret^ with (without) AB/FS mismatch; IDH-mutant_1p/19q^codel^

Quantitatively, the derived CEST metrics with information about amide signal, amine signal, and their ratios enabled the differentiation of IDH-wild-type from IDH-mutant gliomas and the identification of subgroups in the IDH-mutant gliomas (Tables 1, 2, Fig. 4).

**Table 1:** Pairwise comparison of glioma groups for which the CEST metrics identify statistically significant differences. For the ‘two glioma types’, *p*-values were calculated with Mann-Whitney *U* test, whilst for three and four glioma types, *p*-values were calculated with Kruskal-Wallis test followed, when significant, by the Conover-Iman test to correct for multiple pairwise comparisons. AUC is the area under the ROC curve. Threshold values (cut points) for the various metrics are reported together with sensitivity and specificity

Group comparison	Metric	*p*-value	AUC	Cut point	
				Value	Sensitivity/specificity
Two glioma types: IDH-wild-type and IDH-mutant					
IDH-wild-type vs IDH-mutant	AB_APT_w_ (∆2)	0.025	0.74	0.89	0.56/1.00
	FS_APT_w_ (∆2)	0.050	0.71	0.60	0.67/0.67
	AB_APT_w_ ratio	0.0020	0.84	1.57	0.78/0.78
	FS_APT_w_ ratio	0.0045	0.81	1.66	0.78/0.78
	AB-FS (∆2)	0.036	0.73	0.081	0.61/0.78
Three glioma types: IDH-wild-type; IDH-mutant_1p/19q^ret^; and IDH-mutant_1p/19q^codel^					
IDH-wild-type vs IDH-mutant_1p/19q^ret^	AB_APT_w_ (∆2)	0.0020	0.86	0.89	0.77/1.00
	FS_APT_w_ (∆2)	0.014	0.81	0.76	0.68/0.89
	AB_APT_w_ ratio	0.0067	0.85	1.57	0.78/0.82
	FS_APT_w_ ratio	0.021	0.81	1.59	0.89/0.73
	AB-FS (∆3.5)	0.032	0.79	0.048	0.64/0.78
	AB-FS (∆2)	0.0066	0.85	0.081	0.82/0.78
IDH-wild-type vs IDH-mutant_1p/19q^codel^	AB_APT_w_ ratio	0.018	0.79	1.52	0.78/0.69
	FS_APT_w_ ratio	0.022	0.79	1.66	0.78/0.77
IDH-mutant_1p/19q^ret^ vs IDH-mutant_1p/19q^codel^	AB_APT_w_ (∆3.5)	0.035	0.74	1.43	0.55/0.92
	FS_APT_w_ (∆3.5)	0.035	0.74	1.06	0.59/0.85
	AB_APT_w_ (∆2)	0.0027	0.80	0.88	0.77/0.77
	FS_APT_w_ (∆2)	0.015	0.76	0.77	0.68/0.85
	AB-FS (∆3.5)	0.048	0.72	0.029	0.68/0.77
	AB-FS (∆2)	0.015	0.76	0.14	0.68/0.85
Four glioma types: IDH-wild-type; IDH-mutant_1p/19q^ret^ with AB/FS mismatch; IDH-mutant_1p/19q^ret^ without AB/FS mismatch; and IDH-mutant_1p/19q^codel^					
IDH-wild-type vs IDH-mutant_1p/19q^ret^ with AB/FS mismatch	AB_APT_w_ (∆2)	0.0001	0.97	0.89	0.93/1.00
	FS_APT_w_ (∆2)	0.0039	0.90	0.76	0.80/0.89
	AB_APT_w_ ratio	0.0025	0.91	1.39	0.89/0.80
	FS_APT_w_ ratio	0.032	0.85	1.59	0.89/0.80
	AB-FS (∆3.5)	0.0005	0.92	0.067	0.80/0.89
	AB-FS (∆2)	< 0.0001	1.00	0.15	1.00/1.00
	AB-FS_ratio	0.037	0.81	–0.10	0.67/1.00
IDH-wild-type vs IDH-mutant_1p/19q^codel^	AB_APT_w_ ratio	0.035	0.79	1.53	0.78/0.69
IDH-mutant_1p/19q^ret^: with AB/FS mismatch vs without AB/FS mismatch	AB_APT_w_ (∆2)	0.016	0.79	0.82	0.93/0.57
IDH-mutant_1p/19q^ret^ with AB/FS mismatch vs IDH-mutant_1p/19q^codel^	AB_APT_w_ (∆3.5)	0.014	0.81	1.42	0.67/0.92
	FS_APT_w_ (∆3.5)	0.025	0.79	1.28	0.67/0.92
	AB_APT_w_ (∆2)	0.0002	0.90	0.88	0.93/0.77
	FS_APT_w_ (∆2)	0.0049	0.85	0.77	0.80/0.85
	AB-FS (∆3.5)	0.0007	0.85	0.042	0.93/0.85
	AB-FS (∆2)	< 0.0001	0.90	0.14	1.00/0.85
	AB-FS_ratio	0.039	0.76	− 0.16	0.77/0.67

∆3.5 = [3–4 ppm] = 1-ppm frequency range centred on the amide frequency at 3.5 ppm; ∆2 = [1.5–2.5 ppm] = 1-ppm frequency range centred on the amine frequency at 2 ppm; AB_APT_w_ (∆ω) classic CEST metric in the ∆ω range (∆ω = ∆3.5 or ∆ω = ∆2) normalized to the normal appearing white matter; FS_APT_w_ (∆ω) = fluid-suppressed CEST metric in the ∆ω range (∆ω = ∆3.5 or ∆ω = ∆2) normalized to the normal appearing white matter. AB_APT_w_ ratio = AB_APT_w_ (∆3.5)/AB_APT_w_ (∆2); FS_APT_w_ ratio = FS_APT_w_ (∆ω 3.5)/FS_APT_w_ (∆2); AB-FS = AB_APT_w_ − FS_APT_w_

**Table 2:** Median values and 25 to 75 percentile ranges for the asymmetry-based (AB_APT_w_) and fluid-suppressed (FS_APT_w_) normalized CEST metrics, for the various glioma groups and subgroups

Metric	IDH-wild- type Median (25–75 %tiles)	IDH-mutant Median (25–75 %tiles)	IDH-mutant_1p/19q^ret^ Median (25–75 %tiles)	IDH-mutant_1p/19q^codel^ Median (25–75 %tiles)	IDH-mutant_1p/19q^ret^ with AB/FS mismatch Median (25–75 %tiles)	IDH-mutant_1p/19q^ret^ without AB/FS mismatch Median (25–75 %tiles)
AB_APT_w_ (∆3.5)	1.13 (0.79–1.37)	1.04 (0.84–1.61)	1.48 (0.96–1.77)	0.99 (0.61–1.06)	1.67 (0.97–1.82)	1.02 (0.76–1.51)
FS_APT_w_ (∆3.5)	1.07 (0.79–1.32)	1.03 (0.80–1.48)	1.33 (0.90–1.65)	0.98 (0.61–1.03)	1.47 (0.94–1.68)	1.00 (0.76–1.48)
AB_APT_w_ (∆2)	0.59 (0.48–0.75)	0.95 (0.61–1.13)	1.06 (0.92–1.40)	0.64 (0.49–0.85)	1.19 (0.97–1.50)	0.72 (0.38–1.09)
FS_APT_w_ (∆2)	0.56 (0.48–0.75)	0.73 (0.54–0.99)	0.90 (0.63–1.10)	0.59 (0.46–0.64)	0.96 (0.78–1.21)	0.63 (0.38–1.03)
AB_APT_w_ ratio	1.74 (1.59–2.08)	1.30 (1.07–1.53)	1.26 (1.10–1.52)	1.34 (1.03–1.62)	1.21 (1.00–1.39)	1.52 (1.19–1.87)
FS_APT_w_ ratio	1.90 (1.68–2.08)	1.45 (1.22–1.63)	1.44 (1.29–1.62)	1.44 (1.04–1.65)	1.38 (1.14–1.58)	1.52 (1.31–1.98)
AB-FS (∆3.5)	0.003 (0–0.048)	0.032 (0.01–0.12)	0.08 (0.02–0.14)	0.010 (0.000–0.028)	0.12 (0.08–0.20)	0.018 (0–0.022)
AB-FS (∆2)	0.015 (0.005–0.078)	0.12 (0.06–0.23)	0.20 (0.09–0.24)	0.07 (0.00–0.11)	0.22 (0.19–0.38)	0.069 (0–0.086)
AB-FS_ratio	− 0.05 (− 0.18–− 0.04)	− 0.14 (− 0.22–− 0.07)	− 0.15 (− 0.24–− 0.12)	− 0.11 (− 0.16–0)	− 0.19 (− 0.26–− 0.14)	− 0.08 (− 0.13–− 0.01)

∆3.5 = [3–4 ppm] = 1-ppm frequency range centred on the amide frequency at 3.5 ppm; ∆2 = [1.5–2.5 ppm] = 1-ppm frequency range centred on the amine frequency at 2 ppm; AB_APT_w_ (∆ω) asymmetry-based CEST metric in the ∆ω range (∆ω = ∆3.5 or ∆ω = ∆2) normalized to the normal appearing white matter; FS_APT_w_ (∆ω) = fluid-suppressed CEST metric in the ∆ω range (∆ω = ∆3.5 or ∆ω = ∆2) normalized to the normal appearing white matter;. AB_APT_w_ ratio = AB_APT_w_ (∆3.5)/AB_APT_w_ (∆2); FS_APT_w_ ratio = FS_APT_w_ (∆3.5)/FS_APT_w_ (∆2); AB-FS = AB_APT_w_ − FS_APT_w_

The amide/amine signal ratio metrics were higher in IDH-wild-type than in almost all IDH-mutant subgroups (IDH-mutant; IDH-mutant_1p/19q^ret^; IDH-mutant_1p/19q^codel^ and IDH-mutant_1p/19q^ret^ with AB/FS mismatch), but they were not significantly different between IDH-mutant subgroups. IDH-mutant subgroups were, instead, differentiated by amides, amines, and AB-FS metrics.

### AB vs FS metrics and AB/FS mismatch vs T2/FLAIR mismatch

The AB metrics were statistically significantly different from the FS metrics for almost all metrics and glioma subgroup, with the exception of IDH-wild-type in the amides range, and IDH-mutant_1p/19q^ret^ without AB/FS mismatch in the amides range (Table 3).

**Table 3:** Sign test estimates of the differences between AB and FS CEST models for all the investigated metrics: amide signal, amine signal, signal ratio for amides/amines

Group	Metric		
	AB-FS (∆3.5) Median (25–75 %tile) *p*-value	AB-FS (∆2) Median (25–75 %tile) *p*-value	AB-FS_ratio Median (25–75 %tile) *p*-value
NAWM	0.0 (0.0–0.0) 1.0	0.0 (0.0–6.2e-06) 0.21	**-**
IDH-wild-type	0.003 (0–0.048) 0.29	0.015 (0.005–0.078) **0.0078**	− 0.05 (− 0.18–− 0.04) **0.0078**
IDH-mutant	0.032 (0.01–0.12) **< 0.0001**	0.12 (0.06–0.23) **< 0.0001**	− 0.14 (− 0.22–− 0.07) **< 0.0001**
IDH-mutant**_**1p/19q^ret^	0.08 (0.02−0.14) **< 0.0001**	0.20 (0.09–0.24) **< 0.0001**	− 0.15 (− 0.24–− 0.12) **< 0.0001**
IDH-mutant**_**1p/19q^codel^	0.010 (0.0–0.028) **0.0063**	0.07 (0.0–0.11) **0.0018**	− 0.11 (− 0.16–− 0.01) **0.0001**
IDH-mutant**_**1p/19q^ret^ with AB/FS mismatch	0.11 (0.08–0.20) **0.0001**	0.22 (0.19–0.38) **0.0001**	− 0.19 (− 0.25–− 0.13) **0.0001**
IDH-mutant**_**1p/19q^ret^ without AB/FS mismatch	0.018 (0–0.022) 0.22	0.069 (0.001–0.086) **0.016**	− 0.081 (− 0.13–− 0.01) **0.016**

∆3.5 = [3–4 ppm] = 1-ppm frequency range centred on the amide frequency at 3.5 ppm; ∆2 = [1.5–2.5 ppm] = 1-ppm frequency range centred on the amine frequency at 2 ppm; AB-FS = AB − FS models; ratio = amide/amine ratio. *p*-values in bold represent statistically significant values. Threshold for significance *p* = 0.05

A T2/FLAIR mismatch was detected only in IDH-mutant_1p/19q^ret^ gliomas and, more specifically, in 68% (15 out of 22) of them. The difference between AB and FS metrics (AB-FS) for amides and amines (positive differences in both cases, indicating AB > FS values) allowed identifying two subgroups in IDH-mutant_1p/19q^ret^ gliomas: those with and those without AB/FS mismatch. Cut points were calculated for the AB-FS (∆3.5) metric (cut point 0.039, sensitivity 80%, specificity 71%) and AB-FS (∆2) metric (cut point 0.18, sensitivity 80%, specificity 86%) as surrogate biomarkers to assess the presence/absence of T2/FLAIR mismatch, used as a reference standard.

The presence/absence of AB/FS mismatch was in agreement with the presence/absence of T2/FLAIR mismatch, as assessed by a Fisher’s exact test *p*-value of 0.014.

### IDH mutation status

The amines, amide/amine signal ratio, and the difference AB-FS in the amine range differentiated IDH-wild-type from IDH-mutant (Tables 1, 2, Fig. 4). Of these metrics, the amide/amine ratio had the highest AUC (0.84 for AB and 0.81 for FS) and thresholds of 1.57 for AB (1.66 for FS) achieved 78% sensitivity and 78% specificity. In other words, IDH-wild-type had at least a 57% (66% for FS) larger signal coming from the amide component of the CEST spectrum than from the amines.

The AB-FS for amines signal was positive (AB > FS) and larger in IDH-mutant than in IDH-wild-type, suggesting the presence of a larger more ‘fluid’ component in IDH-mutant gliomas (Table 3, Fig. 4).

### IDH mutation and 1p/19q molecular status

When considering IDH mutation and 1p/19q molecular status, significant differences were observed in three pairwise comparisons, after correction for multiple comparisons (Table 1, Fig. 4):

(i) In IDH-wild-type vs IDH-mutant_1p/19q^ret^, the highest AUC was achieved with both the amine signal (0.86 for AB and 0.81 for FS) and the amide/amine signal ratio (0.85 for AB and 0.81 for FS). The thresholds (cut points) were 0.89 (0.76) for the AB (FS) APT_w_ amine (lower amines in IDH-wild-type), with 77% (68%) sensitivity and 100% (89%) specificity; and 1.57 (1.59) for the AB (FS) amide/amine ratio (higher ratio in IDH-wild-type), with 78% (89%) sensitivity and 82% (72%) specificity.
(ii) In IDH-wild-type (the glioma type with the worst prognosis) vs IDH-mutant_1p/19q^codel^ (the glioma type with the best prognosis), only higher amide/amine signal ratio in IDH-wild-type was observed, with both AB and FS models, with thresholds of 1.52 (1.66) for the AB (FS), 78% sensitivity for both AB and FS, and specificity of 69% for AB and 77% for FS.
(iii) In IDH-mutant_1p/19q^ret^ vs IDH-mutant_1p/19q^codel^, the highest AUC (0.80 for AB and 0.76 for FS) was achieved with the amine metrics (higher values in IDH-mutant_1p/19q^ret^), with cut points of 0.88 (0.77) for the AB (FS), sensitivity of 77% for both AB and FS, and specificity of 68% for AB and 85% for FS. Other metrics that differentiated these two subgroups were the amides and the AB-FS differences for both the amides and the amines. The AB amide metric had the highest specificity (92%), but only 55% sensitivity.

### IDH mutation, 1p/19q molecular status, and AB/FS mismatch

When the IDH-mutant_1p/19q^ret^ gliomas were further subdivided in two subgroups (with and without AB/FS mismatch), significant differences were observed in four pairwise comparisons, after correction for multiple comparisons (Table 1, Fig. 4):

(i) In IDH-wild-type vs IDH-mutant_1p/19q^ret^ with AB/FS mismatch, the highest AUC was achieved by the amine signal (0.97 for AB and 0.90 for FS), with thresholds of 0.89 (0.76) for the AB (FS), 93% (80%) sensitivity and 100% (89%) specificity. The amide/amine signal ratio had AUC of 0.91 (0.85) for AB (FS), thresholds of 1.39 (1.59) for the AB (FS), with 89% sensitivity and 80% specificity for both AB and FS.
(ii) In IDH-wild-type (worst prognosis) vs IDH-mutant_1p/19q^codel^ (best prognosis), only higher amide/amine signal ratio in IDH-wild-type were observed with only the AB model, with AUC of 0.79, threshold of 1.53, 78% sensitivity, and 69% specificity.
(iii) In IDH-mutant_1p/19q^ret^ with AB/FS mismatch vs IDH-mutant_1p/19q^ret^ without AB/FS mismatch, only the AB amine metric was higher in IDH-mutant_1p/19q^ret^ with AB/FS mismatch, with AUC of 0.79, threshold of 0.82, sensitivity of 93%, and specificity of 57%. The AB-FS metrics for amides and amines were different by definition (the AB/FS mismatch was defined based on the cut points on these metrics when using the T2/FLAIR mismatch as reference standard).
(iv) In IDH-mutant_1p/19q^ret^ with AB/FS mismatch vs IDH-mutant_1p/19q^codel^, the highest AUC (0.90 for AB and 0.85 for FS) was achieved with the amine-related metrics, with cut points of 0.88 (0.77) for the AB (FS), sensitivity of 93% (80%), and specificity of 77% (85%). Similar AUC were also obtained with AB-FS for amines (AUC 0.90, threshold 0.042, sensitivity 93%, specificity 85%) and AB-FS for amides (AUC 0.85, threshold 0.14, sensitivity 100%, specificity 85%). The amide metrics had the highest specificity (92% for both AB and FS), but only 67% sensitivity (for both AB and FS) and AUC of 0.81 for AB and 0.79 for FS.

No metrics were statistically significant for differentiating IDH-mutant_1p/19q^ret^ without AB/FS mismatch from IDH-mutant_1p/19q^codel^.

## Discussion

The current study demonstrates the potential of CEST metrics using two different models and combining the signal from amides (at 3.5 ppm) and amines (at 2 ppm) to aid in the stratification of gliomas presenting with none or faint post-gadolinium enhancement. Results suggest that (1) the combination of various CEST metrics may be useful biomarkers for differentiating IDH and 1p/19q status; (2) the comparison between the two models (asymmetry-based and fluid-suppressed) may be an objective biomarker to identify the gliomas with T2/FLAIR mismatch.

Other groups report that APT_w_ (amides) alone can differentiate IDH-wild-type/mutant, e.g. [26]. In our study, IDH-wild-type/mutant were differentiated by amines, amide/amine signal ratio, and the difference AB-FS in the amine range, but not by the amide signal. This difference is likely due to the composition of our cohort of diagnostically challenging primary gliomas, in which the IDH-wild-type tumours showed no enhancement in post-contrast T_1_w images (see Supplementary Table 1).

The amide/amine signal ratio was the only metric differentiating IDH-wild type (worst-prognosis) from IDH-mutant_1p/19q^codel^ (best prognosis). The amide/amine signal ratio and levels of amine signal differentiated IDH-wild-type also from the pooled IDH-mutant and from IDH-mutant_1p/19q^ret^. The cut points identified for these comparisons suggested that IDH-wild-type had approximately 60% more signal originating from the amide pool than from amine groups compared to the IDH-mutant groups. IDH-mutant_1p/19q^ret^ had higher amide and amine signal levels than IDH-mutant_1p/19q^codel^. The relevance of tumour acidity in differentiating gliomas with different IDH status is supported by recent studies [25, 40]. Amine and amide signals have been shown to have a complex dependency on protein, amino acids, temperature, and pH concentrations, as well as on CEST saturation [30], whilst the amide/amine signal ratio has been shown to have more straightforward dependency primarily on pH when the hypothesis that amide and amine groups belong to the same molecules is valid (e.g. in ischemia) [31]. The dependency of the CEST amide/amine signal ratio on protein and amino acid concentrations cannot be asserted without additional measurements and simulations. However, the IDH-mutant_1p/19q^ret^ without AB/FS mismatch could not be differentiated from the IDH-mutant_1p/19q^codel^, suggesting that the presence of T2/FLAIR mismatch, usually not assessed in the literature, is an important parameter to consider when assessing the efficiency of the CEST metrics to stratify gliomas.

A complementary imaging technique is amino acid PET (positron emission tomography), an extensively evaluated radiotracer imaging methodology that is playing an increasingly important role in the diagnosis and management of brain tumours. The advantages of amino acid PET are that radiolabelled amino acids cross the blood-brain barrier and their accumulation is a function of tumour avidity for them. This differential uptake can be exploited to specifically delineate cellular mass and tumour boundaries from surrounding normal tissue also in non-enhancing gliomas [41]. A study comparing O-(2-18F-fluoroethyl)-l-tyrosine (FET) PET and APT_w_ CEST MRI in eight high-grade glioma patients showed that FET PET and APT_w_ CEST are spatially incongruent and suggesting different biological information [42]. A further study comparing FET PET with APT_w_ CEST and perfusion (cerebral blood volume, CBV, from a dynamic susceptibility contrast acquisition) in 46 patients (31 IDH-wild-type and 12 IDH-mutant) observed both in IDH-wild-type glioblastomas and IDH-mutant lower grade gliomas relevant overlap between tumour areas defined by different imaging modalities, strongest not only for APT_w_ and FET in contrast-enhancing parts of glioblastomas, but also in the FLAIR-hyperintense region of lower grade gliomas. Furthermore, the author found that both APT_w_ and FET correlated with cellularity, as opposed to CBV which was associated with vascularity [43]. Even though only few studies directly compared amino acid PET and CEST in more than five patients, the complementary and additive values of these methods, as well as their incongruent findings, suggest that further investigation including also neuropathological validation could provide useful information to understand the biological information provided.

Our results also show that the asymmetry-based CEST metric is sensitive to regions with large fluid content (e.g. cystic regions), and that the fluid-suppressed (FS) metric significantly reduces the CEST signal in cysts, without affecting the signal from the normal appearing white matter, but affecting the amide and amine signals in tumour to different extent. We suggest that thresholds can be defined in the differences between the AB and FS models (AB-FS) in the amide and in the amine ranges, to divide the IDH-mutant_1p/19q^ret^ gliomas in two subgroups: with and without AB/FS mismatch (thresholds: AB-FS (∆3.5) = 0.039 and AB-FS (∆2) = 0.18). In IDH-mutant_1p/19q^ret^ glioma, the presence of AB/FS mismatch was closely related to the presence of T2/FLAIR mismatch, suggesting the presence of a more fluid-like compartment (probably micro-cysts, which are observed extracellularly in astrocytomas and intracellularly in oligodendrogliomas) but further studies are needed to explain the factors contributing to our results. As a matter of fact, the presence of AB/FS mismatch and amide/amine signal ratio may be useful biomarkers for differentiating IDH-wild-type from IDH-mutant_1p/19q^ret^ with AB/FS mismatch. However, before any meaningful clinical application may be sought, the biological and prognostic significance of the AB/FS mismatch remains to be validated in larger cohorts. Concerning IDH-mutant gliomas, combinations of amide and amine (but not amide/amine ratio) metrics may be also useful biomarkers for differentiating other glioma subgroups: (i) IDH-mutant_1p/19q^ret^ from IDH-mutant_1p/19q^codel^ and (ii) IDH-mutant_1p/19q^ret^ with AB/FS mismatch from IDH-mutant_1p/19q^ret^ without AB/FS mismatch.

In our data, the amine range had at times a higher sensitivity than the amide range to distinguish between tumour types. Several groups have investigated the contributions to the CEST signal at 2 ppm showing that the major contributions, at pH = 7 and at a temperature of 37 °C, come from creatine (Cr), glutamate (Glu, roughly 40% of Cr signal), phosphocreatine (PCr), ATP (both approximately 20% of Cr signal), glucose (Glc, roughly 10% of Cr signal), and proteins [14], with various weightings of these different pools depending on the CEST saturation scheme and other MRI acquisition parameters. Some have suggested that at this pH and temperature, glutamine (Gln) does not contribute to the CEST signal at 2 ppm [14], while others have found contribution from Gln, especially in tumours with a more acidic extracellular microenvironment [44]. It has also been suggested that also direct water saturation, semi-solid MT, and water longitudinal relaxation time effects, which contribute to the signal non-linearly, are likely to contribute to the CEST signal at 2 ppm [14]. It is possible that the presence of all these compounds at 2 ppm increases the ability of the CEST signal at 2 ppm to differentiate between tumour types. However, contributions from the nuclear Overhauser enhancement (NOE)–mediated effect coming from the upfield resonances complicate the interpretations of our results, and it may contribute to the low sensitivity of the CEST amide signal for distinguishing between tumour types. The MTR_asym_ offset dependence is due to the presence of saturation peaks in a frequency range from − 1 to – 5 ppm, mediated by the NOE. The NOE is therefore entangled to the chemical exchange effect in the MTR_asym_ measure of the magnetization transfer process. A positive MTR_asym_ reflects a predominant chemical exchange effect while a negative MTR_asym_ reflects a predominant NOE-mediated effect. Limitations of the present study include the presence of the NOE-mediated effects in the MTR_asym_ and ∆MTR_asym_ spectra. The NOE-mediated contribution is reduced with increasing *B*_1_ saturation power and saturation powers of 2μT had been shown in the past to null the NOE-mediated effect at 3.5 ppm [45, 46]. Our normal appearing white matter MTR_asym_ signal nulls at 3 ppm from the water resonance (Fig. 3a), indicating that the balance between CEST and NOE effects was obtained at 3 ppm and there was a slightly predominant NOE contribution for frequencies above 3 ppm [45, 46].

Our data acquisition was also limited to a single slice. Since the start of our study, 3D CEST sequences have become available at both 3T and 7T [47, 48] together with more complex analysis methods based on fitting multiple Lorentzian shapes to the data allowing the quantification of the different contributions from amides, amines, NOE, MT, and water [32, 48]. We plan to introduce these acquisition and analysis strategies in our future studies, together with MRS and perfusion and diffusion measurements to further characterize gliomas both pre- and post-treatments.

## Conclusions

The current study suggests that CEST-derived biomarkers for amines and amides, together with their ratio, which reflects in a composite manner tissue acidity and proteins/amino acid abundance, can be used for histomolecular staging in a cohort of diagnostically challenging non- or low-enhancing gliomas. The data also suggests that the mismatch between CEST maps obtained with different models (asymmetry-based (AB) and fluid-suppressed (FS)) can be used to identify subgroups in IDH-mutant_1p/19q^ret^ gliomas that could potentially have prognostic and clinical relevance. CEST-derived biomarkers could therefore serve as risk stratification tools to inform oncologists of recurrence risk or of optimal treatment.

## Supplementary Information

Below is the link to the electronic supplementary material.

Supplementary file1 (DOCX 57.5 KB)

## Data Availability

The authors confirm that the data supporting the findings of this study are available within the article, in its Supplementary Material and/or from the corresponding author upon reasonable request.

## Abbreviations

1p/19q^ret^: 1p/19q retained
1p/19q^codel^: 1p/19q codeleted
AB: Asymmetry-Based
APT_w_: Amine Proton Transfer-weighted
AUC: Areas Under the receiver operating characteristic Curve
CEST: Chemical Exchange Saturation Transfer
DWI: Diffusion-Weighted Imaging
GBM: Glioblastoma Multiforme
∆MTR_asym_: Changes in MTR_asym_
FS: Fluid-Suppressed
IDH: Isocitrate DeHydrogenase
MRS: Magnetic Resonance Spectroscopy
MTR_asym_: Magnetization Transfer Ratio asymmetry
NAWM: Normal Appearing White Matter
NOE: Nuclear Overhauser Enhancement
ROE: Receiver Operating Characteristics
