# Correction to: CEST MRI provides amide/amine surrogate biomarkers for treatment‑naïve glioma sub‑typing

**DOI:** 10.1007/s00259-022-05798-6

**Published:** 2022-04-12

**Authors:** Laura Mancini, Stefano Casagranda, Guillaume Gautier, Philippe Peter, Bruno Lopez, Lewis Thorne, Andrew McEvoy, Anna Miserocchi, George Samandouras, Neil Kitchen, Sebastian Brandner, Enrico De Vita, Francisco Torrealdea, Marilena Rega, Benjamin Schmitt, Patrick Liebig, Eser Sanverdi, Xavier Golay, Sotirios Bisdas

**Affiliations:** 1 Box65, Lysholm Department of Neuroradiology, The National Hospital for Neurology & Neurosurgery, University College London Hospitals NHS Foundation Trust, 8‑11 Queen Square, London, WC1N 3BG UK; 2 Department of Brain Repair and Rehabilitation, UCL Queen Square Institute of Neurology, London, UK; 3 Olea Medical, La Ciotat, France; 4 Department of Neurosurgery, The National Hospital for Neurology & Neurosurgery, University College London Hospitals NHS Foundation Trust, London, UK; 5 Division of Neuropathology, UCL Queen Square Institute of Neurology, London, UK; 6 The National Hospital for Neurology and Neurosurgery, University College London Hospitals NHS Foundation Trust, London, UK; 7 University College Hospital, University College of London Hospitals NHS Foundation Trust, London, UK; 8 Siemens Healthineers, Erlangen, Germany

**Correction to: Eur J Nucl Med Mol Imaging**

**https://doi.org/10.1007/s00259-022-05676-1**

The authors regret that the version of Figure 3 that appears in the original article is incorrect.

The original article has been corrected.

