# Supplementary material for: CEST MRI provides amide/amine surrogate biomarkers for treatment-naïve glioma sub-typing
Source: Eur J Nucl Med Mol Imaging. 2022 Jan 14;49(7):2377–91. doi: 10.1007/s00259-022-05676-1 (PMC9165287; doi:10.1007/s00259-022-05676-1)
Supplement: Supplementary file 1 — Supplementary file1 (DOCX 57.5 KB) [file 259_2022_5676_MOESM1_ESM.docx]

# **Supplementary material for:**

European Journal of Nuclean Medicine and Molecular Imaging

**CEST MRI provides amides/amines surrogate biomarkers for treatment-naïve glioma sub-typing**

Laura Mancini^1,2^, Stefano Casagranda^3^, Guillaume Gautier^3^, Philippe Peter^3^, Bruno Lopez^3^, Lewis Thorne^6^, Andrew McEvoy^6^, Anna Miserocchi^6^, George Samandouras^6^, Neil Kitchen^6^, Sebastian Brandner^4,5^, Enrico De Vita^1,2^, Francisco Torrealdea^7^, Marilena Rega^7^, Benjamin Schmitt^8^, Patrick Liebig^8^, Eser Sanverdi^1,2^, Xavier Golay^1,2^, and Sotirios Bisdas^1,2^

**Author affiliations:**

1 Lysholm Department of Neuroradiology, The National Hospital for Neurology & Neurosurgery, University College London Hospitals NHS Foundation Trust, London, UK

2 Department of Brain Repair and Rehabilitation, UCL Queen Square Institute of Neurology, London, UK

3 Olea Medical, La Ciotat, France

4 Division of Neuropathology, UCL Queen Square Institute of Neurology

5 The National Hospital for Neurology and Neurosurgery, University College London Hospitals NHS Foundation Trust, London, UK

6 Department of Neurosurgery, The National Hospital for Neurology & Neurosurgery, University College London Hospitals NHS Foundation Trust, London, UK

7 University College Hospital, University College of London Hospitals NHS Foundation Trust, London, UK

8 Siemens Healthineers, Erlangen, Germany

Correspondence to: Laura Mancini

Box65, Lysholm Department of Neuroradiology, The National Hospital for Neurology and Neurosurgery, University College of London Hospitals NHS Foundation Trust,

8-11 Queen Square, WC1N 3BG, London, UK

E-mail: [lmancini@nhs.net](mailto:lmancini@nhs.net)

ORCID ID: 0000-0002-4596-3551

## **Parameters of the MRI Data Acquisitions**

### **Structural Images**

Structural T_1_-weighted (T_1_w), T_2_w, fluid attenuated inversion recovery (FLAIR) and gadolinium-enhanced T_1_w (Gd-T_1_w) images were acquired with sagittal 3D SPACE images with GRAPPA acceleration factor=2, 3D acceleration factor=2 and the following parameters. T_1_w and Gd-T_1_w: FOV = 230x230 mm^2^, acquisition matrix = 256x256, one slab of 192 slices with 0.9 mm slice thickness, TR/TE = 700/11 ms, flip angle mode = T_1_var, echo train duration (ETD) = 155 ms, turbo factor (TF) = 38, BW = 630 Hz/px and TA = 4 min: 23 s. T_2_w: FOV = 282x282 mm^2^, acquisition matrix = 256x256, one slab of 176 slices with 1.1 mm slice thickness, TR/TE = 3200/401 ms, flip angle mode = T_2_var, ETD = 872ms, TF = 282, BW = 751 Hz/px and TA = 3 min: 49 s. FLAIR: FOV = 250x250 mm^2^, acquisition matrix = 256x256, one slab of 176 slices with 1.0 mm slice thickness, TR/TE = 5000/502 ms, magnetisation preparation = non-selective T_2_-IR, inversion time (TI) = 1600 ms, flip angle mode = T_2_var, ETD = 1017 ms, TF = 300, BW = 751 Hz/px and TA = 4 min: 22 s.

### **CEST acquisitions**

Three 2D single-slice CEST acquisitions were performed with B_1_ values = 1.7 μT, 2.0 μT and 2.3 μT, to allow for B_1_ inhomogeneity correction. Saturation was obtained with a train of 41 Gaussian-shaped RF pulses of pulse duration *τ*_p_ = 44.8 ms with inter-pulse delay *τ*_d_ = 40 ms (duty cycle = 53%, saturation time = 3.48 s, flip angle of the saturation pulses calculated with the continuous wave power equivalent (CWPE) $\omega_{1}=\sqrt{\frac{1}{\tau_{p} + \tau_{d}}\int_{0}^{\tau_{p}} \omega_{1}^{2}\left( t \right)\mathrm{dt}}$ $\omega_{1}=\sqrt{\frac{1}{\tau_{p}+\tau_{d}}\int_{0}^{\tau_{p}} \omega_{1}^{2}\left( t \right)\mathrm{dt}}$. Spoiling with variable amplitudes and polarities on each axis after the saturation pulses was applied to minimize the occurrence of stimulated echoes. An initial normalisation image was acquired with pre-saturation at Δω = −150 ppm from water resonance frequency, to suppress the macromolecular background. This was followed by 37 equally spaced alternating negative/positive offsets, from ±4.5 ppm to 0 ppm in steps of 0.25 ppm. 1 ppm equals to 123 Hz for this specific 3T scanner. Other image parameters were: field of view (FOV) = 210x170 mm^2^, acquisition matrix = 128x104, slice thickness = 4 mm, repetition time (TR)/echo time (TE) = 8.2/3.28 ms, flip angle = 15°, bandwidth (BW) = 150 Hz/px and acquisition time (TA) for each of the three B_1_ values = 2 min: 43 s.

A flip angle map, used to compute a relative B1 map [1] was acquired using a turbo Fast Low Angle SHot (FLASH) sequence, with acquisition parameters: FOV = 210x170 mm^2^, acquisition matrix = 128x104, one slice with 4 mm slice thickness, TR/TE = 4350/2.06 ms, nominal flip angle = 8°, TF = 136, BW = 490 Hz/px and TA = 8.7 s.

## **CEST post-processing**

### **Asymmetry-Based and Fluid-Suppressed APTw Image Processing**

The AB_APT_w_ considered only the asymmetry-average of the Z-Spectra in a specific offset range:

Equation 1: AB_APT_w_ = $\frac{\int_{-\Delta\omega2}^{-\Delta\omega1} \tilde{Z}\left( \Delta\omega\right)d\omega-\int_{\Delta\omega1}^{\Delta\omega2} \tilde{Z}\left( \Delta\omega\right)d\omega}{\Delta\omega2-\Delta\omega1}$ (1)

While the FS_APT_w_ also attenuates the fluid signal based on the shape of Z-spectrum:

Equation 2: FS_APT_w_ = $\frac{\int_{-\Delta\omega2}^{-\Delta\omega1} \tilde{Z}\left( \Delta\omega\right)d\omega-\int_{\Delta\omega1}^{\Delta\omega2} \tilde{Z}\left( \Delta\omega\right)d\omega}{\Delta\omega2-\Delta\omega1}\cdot(1-\frac{\int_{-\Delta\omega2}^{-\Delta\omega1} \tilde{Z}\left( \Delta\omega\right)d\omega}{\Delta\omega2-\Delta\omega1})\cdot2\alpha$ (2)

where $\tilde{Z}$is the linear-interpolated Z-Spectrum, $\Delta\omega_{1}, \Delta\omega_{2}$ the offsets and *α* a regularisation parameter carefully selected to avoid altering the NAWM signal between the asymmetry-based and fluid-suppressed metrics (p values for the differences between the two models in NAWM for the amides and amines regions were, respectively, 1.0 and 0.21, Supplementary Table 2).

The endogenous CEST signal was explored in two offset ranges: the amines ($\Delta2=\Delta\omega_{2}-\Delta\omega_{1}$, with $\Delta\omega_{1}$ = 1.5 ppm and $\Delta\omega_{2}$= 2.5 ppm, α = 0.75) and the amides ones ($\Delta3.5=\Delta\omega_{2}-\Delta\omega_{1}$, $\Delta\omega_{1}$ = 3.0 ppm, $\Delta\omega_{2}$= 4.0 ppm, α = 1), obtaining four maps: AB_APT_w_(Δ2) and AB_APT_w_(Δ3.5) calculated using Equation 1, and FS_APT_w_(Δ2) and FS_APT_w_(Δ3.5) using Equation 2. The average values of the four maps in the tumour were normalised by subtracting from their values the average signal in the contralateral NAWM ROIs. In total, 9 normalised metrics were considered: (i-ii) the amides, AB_APT_w_(Δ3.5) and FS_APT_w_(Δ3.5); (iii-iv) the amines, AB_APT_w_(Δ2) and FS_APT_w_(Δ2); (v-vi) the amides/amines ratios AB_APT_w_ratio = AB_APT_w_(Δ3.5)/AB_APT_w_(Δ2) and FS_APT_w_ratio = FS_APT_w_(Δ3.5)/FS_APT_w_(Δ2); (vii-ix) the differences between the asymmetry-based and the fluid-suppressed models for all of the above metrics: AB–FS(Δ3.5) = AB_APT_w_(Δ3.5)–FS_APT_w_(Δ3.5); AB–FS(Δ2) = AB_APT_w_(Δ2)–FS_APT_w_(Δ2); AB–FS_ratio = AB_APT_w_ratio–FS_PT_w_ratio.

## **References**

1. Windschuh J, Zaiss M, Meissner JE, Paech D, Radbruch A, Ladd ME, et al. Correction of B1-inhomogeneities for relaxation-compensated CEST imaging at 7T. NMR in Biomedicine; 2015. p. 529-37.

Supplementary Table 1 Glioma patient cohort included in this study

| **Patient** | **Sex** | **Age (years)** | **IDH** | **1p/19q** | **Hist. App. Grade** | **Integrated Grade** | **Integrated Diagnosis** | **Gd Enhancement** |
| --- | --- | --- | --- | --- | --- | --- | --- | --- |
| #1 | F | 24.0 | mut | codel | II | II | OII_IDH | No |
| #2 | M | 53.4 | mut | codel | III | III | OIII_IDH | No |
| #3 | M | 62.7 | wt | ret | II | IV | GBM_WT | No |
| #4 | M | 22.7 | mut | ret | III | III | AIII_IDH | No |
| #5 | M | 40.7 | mut | ret | II | II | AII_IDH | No |
| #6 | F | 33.8 | mut | codel | II | II | OII_IDH | No |
| #7 | F | 68.7 | wt | ret | III | III | AIII_WT | Faint |
| #8 | F | 56.1 | mut | codel | III | III | OIII_IDH | Faint |
| #9 | F | 26.5 | mut | ret | III | III | AIII_IDH | No |
| #10 | F | 45.1 | mut | ret | II | II | AII_IDH | No |
| #11 | F | 63.1 | wt | ret | II | IV | GBM_WT | No |
| #12 | F | 39.5 | mut | ret | II | II | AII_IDH | No |
| #13 | M | 26.9 | wt | ret | III | III | AIII_WT | Faint |
| #14 | M | 39.4 | mut | codel | II | II | OII_IDH | No |
| #15 | F | 56.6 | mut | codel | II | II | OII_IDH | No |
| #16 | F | 36.8 | mut | codel | II | II | OII_IDH | No |
| #17 | M | 56.1 | wt | ret | II | IV | GBM_WT | No |
| #18 | F | 60.1 | wt | ret | III | IV | GBM_WT | No |
| #19 | M | 24.5 | mut | ret | II | II | AII_IDH | No |
| #20 | M | 45.9 | mut | ret | II | II | AII_IDH | No |
| #21 | M | 41.1 | mut | ret | II | II | AII_IDH | No |
| #22 | F | 49.5 | mut | codel | II | II | OII_IDH | No |
| #23 | M | 28.6 | mut | ret | II | II | AII_IDH | No |
| #24 | F | 24.6 | mut | ret | II | II | AII_IDH | No |
| #25 | M | 40.9 | mut | ret | II | II | AII_IDH | Moderate |
| #26 | F | 75.7 | mut | codel | II | II | OII_IDH | No |
| #27 | F | 56.2 | mut | codel | II | II | OII_IDH | No |
| #28 | F | 38.7 | mut | ret | II | II | AII_IDH | Faint |
| #29 | F | 36.9 | mut | ret | II | II | AII_IDH | Faint |
| #30 | M | 34.3 | mut | ret | II | II | AII_IDH | Faint |
| #31 | F | 36.4 | mut | ret | IV | IV | GBM_IDH | No |
| #32 | M | 47.9 | mut | codel | II | II | OII_IDH | No |
| #33 | M | 35.8 | mut | codel | II | II | OII_IDH | No |
| #34 | F | 34.6 | mut | ret | III | III | AIII_IDH | Moderate |
| #35 | M | 32.8 | mut | ret | II | II | AII_IDH | No |
| #36 | M | 46.4 | mut | codel | II | II | OII_IDH | No |
| #37 | F | 33.7 | mut | codel | II | II | OII_IDH | No |
| #38 | F | 41.1 | mut | ret | II | II | AII_IDH | Faint |
| #39 | F | 26.1 | mut | ret | III | III | AIII_IDH | No |
| #40 | F | 36.6 | mut | ret | II | II | AII_IDH | No |
| #41 | F | 30.1 | mut | ret | II | II | AII_IDH | No |
| #42 | M | 33.4 | mut | ret | IV | IV | GBM_IDH | No |
| #43 | M | 57.5 | wt | ret | II | IV | GBM?_WT* | No |
| #44 | F | 52.7 | mut | codel | n/a | III | OIII_IDH | No |
| #45 | F | 65.8 | wt | ret | n/a | n/a | A?_WT** | No |
| AII_IDH = astrocytoma grade II IDH-mutant, AIII_IDH = astrocytoma grade III IDH-mutant, AIII_WT = astrocytoma grade III IDH-wildtype, OII_IDH = oligodendroglioma grade II IDH-mutant, OIII_IDH = oligodendroglioma grade III IDH-mutant, GBM_IDH = glioblastoma IDH-mutant, GBM_WT = glioblastoma IDH-wildtype. * Molecular profile suggestive of GBM. ** Morphological appearances suggestive of an infiltration of an astrocytoma. Hist. App. = Histological Appearance. | | | | | | | | |
